# Supplementary material for: Inferring clonal composition from multiple tumor biopsies
Source: NPJ Syst Biol Appl. 2020 Aug 25;6:27. doi: 10.1038/s41540-020-00147-5 (PMC7447821; doi:10.1038/s41540-020-00147-5)
Supplement: Supplementary file 1 — Supplementary Material [file 41540_2020_147_MOESM1_ESM.pdf]

SUPPLEMENTARY TABLES

All supplementary tables are accessible through the Chimæra GitHub repository, branch journal-submission (https://github.com/drugilsberg/chimaera/tree/journal-submission).

Table S1. Synthetic tree descriptions.

Table S2. Analysis of all subsets of regions in profiled tumors; associated with Figure 4.

Table S3. Data and Analysis of all HCC mutations.

Table S4. Data and Analysis of all Wilms' tumor profiles.

Table S5. Data and Analysis of all prostate cancer profiles.
